# A novel cuproptosis-related prognostic 2-lncRNAs signature in breast cancer

**DOI:** 10.3389/fphar.2022.1115608

**Published:** 2023-01-09

**Authors:** Qi-Tong Xu, Zi-Wen Wang, Meng-Yuan Cai, Ji-Fu Wei, Qiang Ding

**Affiliations:** ^1^ Jiangsu Breast Disease Center, The First Affiliated Hospital with Nanjing Medical University, Nanjing, China; ^2^ Department of Pharmacy, Jiangsu Cancer Hospital, Jiangsu Institute of Cancer Research, The Affiliated Cancer Hospital of Nanjing Medical University, Nanjing, China

**Keywords:** breast cancer, cuproptosis, long-noncoding RNA (LncRNA), prognostic signature, bioinformatics, machine learning

## Abstract

**Background:** Cuproptosis, a newly defined regulated form of cell death, is mediated by the accumulation of copper ions in cells and related to protein lipoacylation. Seven genes have been reported as key genes of cuproptosis phenotype. Cuproptosis may be developed by subsequent research as a target to treat cancer, such as breast cancer. Long-noncoding RNA (lncRNA) has been proved to play a vital role in regulating the biological process of breast cancer. However, the role of lncRNAs in cuproptosis is poorly studied.

**Methods:** Based on TCGA (The Cancer Genome Atlas) database and integrated several R packages, we screened out 153 cuproptosis-related lncRNAs and constructed a novel cuproptosis-related prognostic 2-lncRNAs signature (BCCuS) in breast cancer and then verified. By using pRRophetic package and machine learning, 72 anticancer drugs, significantly related to the model, were screened out. qPCR was used to detect the differentially expression of two model lncRNAs and seven cuproptosis genes between 10 pairs of breast cancer tissue samples and adjacent samples.

**Results:** We constructed a novel cuproptosis-related prognostic 2-lncRNAs (USP2-AS1, NIFK-AS1) signature (BCCuS) in breast cancer. Univariate COX analysis (*p* < .001) and multivariate COX analysis (*p* < .001) validated that BCCuS was an independent prognostic factor for breast cancer. Overall survival Kaplan Meier-plotter, ROC curve and Risk Plot validated the prognostic value of BCCuS both in test set and verification set. Nomogram and C-index proved that BCCuS has strong correlation with clinical decision-making. BCCuS still maintain inspection efficiency when patients were splitting into Stage I−II (*p* = .024) and Stage III−IV (*p* = .003) breast cancer. BCCuS-high group and BCCuS-low group showed significant differences in gene mutation frequency, immune function, TIDE (tumor immune dysfunction and exclusion) score and other phenotypes. TMB (tumor mutation burden)-high along with BCCuS-high group had the lowest Survival probability (*p* = .005). 36 anticancer drugs whose sensitivity (IC50) was significantly related to the model were screened out using pRRophetic package. qPCR results showed that two model lncRNAs (USP2-AS1, NIFK-AS1) and three Cuproptosis genes (FDX1, PDHA1, DLAT) expressed differently between 10 pairs of breast cancer tissue samples and adjacent samples.

**Conclusion:** The current study reveals that cuproptosis-related prognostic 2-lncRNAs signature (BCCuS) may be useful in predicting the prognosis, biological characteristics, and appropriate treatment of breast cancer patients.

## Introduction

Breast cancer has become the most common malignant tumor in women worldwide, with the incidence rate continuing to increase (Harbeck and Gnant, 2017; Bray et al., 2018; DeSantis et al., 2019). It is classified into luminal type A, Luminal type B, HER-2 overexpression, basal like type and other special types (Sotiriou and Pusztai, 2009; Goldhirsch et al., 2011; Prat and Perou, 2011). The prognosis and treatments mainly depend on the stage and subtype of breast cancer. Among them, triple negative breast cancer lacks effective therapeutic targets, which often shows a relatively inferior prognosis (Rakha et al., 2008; Waks and Winer, 2019). A prognostic factor can independently predict the outcome of cancer (disease recurrence, disease progression or death), but do not have significant correlation with the treatment received, so it can help to make a clinical decision (Yu et al., 2019). For example, CLEOPATRA (Clinical Evaluation of Pertuzumab and Trastuzumab) test proved that the mutate of PIK3CA was a prognostic factor for patients with advanced HER2 positive breast cancer. In both the control group and the treatment group, the PFS (progression free survival) of patients with PIK3CA mutate is significantly worse than patients with PIK3CA wild type (Giordano et al., 2014; Swain et al., 2020). However, the complexity of breast cancer determines that even in its early stage, it is difficult to diagnose and predict the prognosis through a single protein or gene like the standard clinicopathological predictors (Fumagalli and Sotiriou, 2010). At present, there is no reliable single marker that can detect the prognosis of breast cancer and predict the effect of drug therapy (Paoletti and Hayes, 2014). Several multiple molecules signature to predict the prognosis of breast cancer and assisting clinical decision, such as BCI (breast cancer index), Oncotype DX and MammaPrint (Mathieu et al., 2012). Breast cancer 21 gene detection (Oncotype DX) includes 16 breast cancer related genes (proliferation group, invasion group, estrogen group, etc.,) and five reference genes, and calculate the recurrence score (RS) according to the expression level of 21 genes and provide chemotherapy effect prediction and 10-year recurrence risk assessment (Cronin et al., 2007; Gyanchandani et al., 2016). Breast cancer 70 gene detection (MammaPrint) is a detection product mainly used to assess the risk of distant metastasis of patients within 5 years (Mook et al., 2010; Tsai et al., 2018). Although there exist mature multi-gene prognostic targets for breast cancer, simpler models based on new phenotypes still need to be developed.

Cuproptosis is a newly defined form of cell death that is mediated by the accumulation of copper ions in cells, which is obviously different from the already known cell apoptosis, pyrosis, necroptosis and ferroptosis (Tsvetkov et al., 2022). It occurs through the direct combination of copper and the fatty acylated components of the tricarboxylic acid cycle (TCA) (Tsvetkov et al., 2022). It leads to the aggregation of fatty acylated proteins and the loss of iron sulfur cluster proteins, which in turn triggers proteotoxic stress and ultimately leads to cell death. By the genome-wide CRISPR-cas9 screening, seven genes (FDX1, LIPT1, LIAS, DLD, DLAT, PDHA1, PDHB) were found to be related to cuproptosis (Tsvetkov et al., 2022). The abundance of FDX1 and lipoacylated proteins are highly correlated with a variety of human tumors. Breast cancer cell lines with high levels of lipoacylated proteins were shown to be more sensitive to accumulation of copper ions (Manikandamathavan et al., 2017; Shanbhag et al., 2019). In case of mutation, LIAS can convert HIF1-α stable in non-hydroxylated form, HIFI-α activation is the basis of the abnormal functional switch of EZH2/PRC2 in breast cancer (Burr et al., 2016; Mahara et al., 2016). KIAA1735 gene and DLAT gene are linked in a tail to head manner, and KIAA1735 gene is deleted in breast cancer (Katoh and Katoh, 2003). Oncoprotein HBXIP enhances glucose metabolism reprogramming by inhibiting PDHA1 in breast cancer (Liu et al., 2015). PDHB has been reported to be associated with high PRA: PRB in mammary gland of transgenic mice in breast cancer, which indicates breast cancer malignant progression (Carlini et al., 2018). These results suggests that copper ionophore may be a potential “silver bullet” for breast cancer cells.

Long-noncoding RNA (lncRNA) are closely related to copper ion accumulation, short term inhalation of copper welding fume can lead to the increase of levels of four lncRNAs: CoroMarker, MALAT1, CDR1as and LINC00460 (Scheurer et al., 2022). Cuproptosis-related lncRNA can predict prognosis of sarcoma, gastric cancer, and renal cell carcinoma (Xu et al., 2022a; Feng et al., 2022; Yang et al., 2022). Long-noncoding RNA (lncRNA) also has been proved to play a key role in regulating the biological process of breast cancer. LncRNA-BCRT1 can competitively bind with mir-1303 to reduce the degradation of its downstream gene PTBP3, which plays a cancer promoting role in breast cancer (Liang et al., 2020). LncRNA-H19 induces autophagy activation through the H19/SAHH/DNMT3b pathway, which may affect the resistance of breast cancer to tamoxifen (Wang et al., 2019). Other researchers found that LncRNA-SCRIT can inhibit the transcriptional activity of EZH2 through direct interaction with EZH2 and regulate the proliferation and invasion of breast cancer cells (Pardini and Dragomir, 2021).

Considering the significance of lncRNA and cuproptosis in breast cancer, we constructed a novel cuproptosis-related prognostic 2-lncRNAs signature (BCCuS) to predict the prognosis, biological characteristics, and appropriate treatment of breast cancer patients.

## Materials and methods

### Process summary and data sources

**FIGURE 1:**
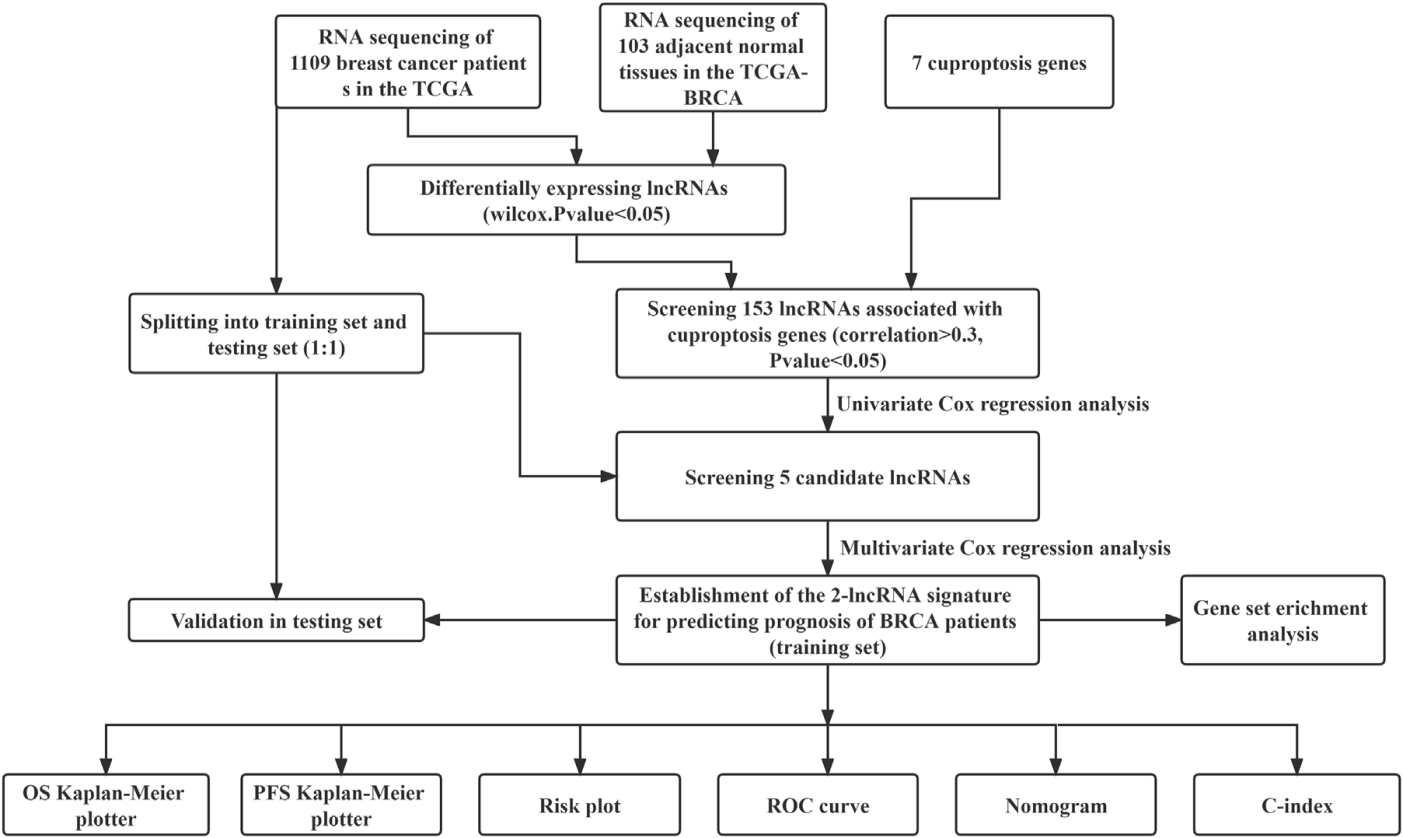
Flow chart for identification and verification of cuproptosis-related prognostic 2-lncRNAs signature (BCCuS) in TCGA breast cancer patients. qPCR was used to detect the differentially expression of two model lncRNAs and seven cuproptosis genes between 10 pairs of breast cancer tissue samples and adjacent samples.

The workflow of our analysis was shown in Figure 1. The gene expression RNAseq (HTSeq-FPKM), clinicopathological data, survival data, mutation data, breast cancer were downloaded from the online database TCGA(https://cancergenome.nih.gov). The data of TCGA breast cancer patients were randomly divided into training set and internal verification set at a ratio of 1:1. Chi square test verified the fairness of grouping.

### Identification of cuproptosis-related lncRNAs

According to gencode (https://www.gencodegenes.org), lncRNAs was extracted. Seven cuproptosis-related genes including FDX1, LIPT1, LIAS, DLD, DLAT, PDHA1 and PDHB (Tsvetkov et al., 2022). Pearson method was used to analyze the correlation between lncRNAs and cuproptosis-related genes in breast cancer. 153 LncRNAs with correlation coefficient R2 > .3 and *p* < .05 were considered as cuproptosis-related lncRNAs. The expression data of these 153 lncRNAs were extracted from the breast cancer data of TCGA. Then the data were equally divided into training set (*n* = 555) and internal verification set (*n* = 554). All RNA sequencing data were normalized by log2 conversion.

### lncRNAs model constructed

Univariate Cox regression analysis was used to obtain lncRNAs associated with prognosis from the training dataset. LncRNAs with *p* < .05 were then included in multivariate Cox risk model analysis to generate cuproptosis-related lncRNA model. To avoid the model being too complicated, Akaike information criterion (AIC) was used to evaluate the complexity of the model, and model with lowest AIC value (AIC = 695.15) and no reduction in prediction efficiency was evaluated as best. The risk score of each breast cancer patient was calculated according to the following formula: BCCuS (risk score) = *γ*1 × expression (lncRNA1)+ *γ*2 × expression (lncRNA2) + … + γn × expression (lncRNAn). γn is the regression coefficient of the corresponding lncRNA, expression (lncRNAn) is the expression level of lncRNA, and the unit is FPKM. According to the median of risk score, 555 breast cancer patients in the training data set were divided into two groups: high-risk group and low-risk group. To compare OS differences between high-risk and low-risk groups, Kaplan Meier plotter analysis was implemented. In addition, the predictive ability of the model was evaluated by performing ROC curves using the survivalROC R package. The diagnostic value of BCCuS was further validated by Risk Plot analysis by using pheatmap package. Finally, the expression and prognostic data of 554 patients in the test set were used to test the efficacy of the model.

### Model test and mechanism exploration

Nomogram and C-index was used to prove that BCCuS has strong correlation with clinical outcomes and can tutor decision-making. Then patients were splitting into Stage I-II and Stage III-IV; Age < 60 and Age > 60, BCCuS still maintain inspection efficiency. TMB (tumor mutation burden) data and base mutation data of breast cancer samples were downloaded from TCGA database. Breast cancer samples were divided into high and low groups according to the median risk scores, and the frequency of gene mutations and TMB in the two groups was calculated by using maftools package. GSVA and limma package was used to analyze the differences in immune function between BCCuS-high and BCCuS-low risk groups. We also evaluated the relationship between BCCuS and tumor immune dysfunction and exclusion (TIDE) score (https://tide.dfci.harvard.edu/).

### Screening of anticancer drugs

By using pRRophetic package and machine learning, 36 anticancer drugs whose sensitivity (IC50) was significantly related to the model were screened out. The threshold of *p*-value was .001, and the anticancer drug sensitivity database used was CPG 2016, which was included in pRRophetic package.

### Acquisition of breast tissue samples

We used the primary breast cancer tissues and normal tissues adjacent to the cancer from 10 patients with breast cancer diagnosed in the breast center of Jiangsu Province, China. The deadline for follow-up was April 2022. All patients provided written informed consent. This was done in accordance with the declaration of Helsinki. All samples were obtained with the approval of the hospital ethics committee.

### Quantitative real-time PCR (qRT-PCR)

Total RNA was isolated from tissues and cells using Trizol reagent (Invitrogen, United States) according to the manufacturer’s protocol. CDNA was synthesized using hiscript II (vazyme, China). Then, qRT-PCR of mRNA and lncRNA was performed on a stepone plus real-time PCR system (Applied Biosystems, United States). U6 and β- Actin was used as a standard control for lncRNA and mRNA detection, respectively. The gene expression in PCR was obtained by logarithmic conversion of CT value. All PCR primers (lifetech, China) are listed in Table 1.

**TABLE 1:** All PCR primers.

Primer name	Primer sequence (5′—3′)
PDHBF	aga​gga​cac​gac​caa​gat​gg
PDHBR	ttc​cac​agc​cct​cga​cta​ac
PDHA1F	gac​tgt​acg​ccg​aat​gga​gt
PDHA1R	ggg​tga​aag​taa​agc​cgt​ga
LIASF	cga​gat​gat​atg​cct​gat​gg
LIASR	cga​agt​gct​ttc​att​gtt​gc
FDX1F	ctg​tcc​tga​gct​gga​gaa​gg
FDX1R	tgg​taa​tct​gtg​gtg​ctt​gc
DLDF	aga​tgg​cat​ggt​gaa​gat​cc
DLDR	cca​aat​gac​gca​gca​aga​tt
DLATF	gac​caa​agg​gaa​ggg​tgt​tt
DLATR	cgg​agc​agg​agc​aac​ttt​ac
NIFK-AS1F	tgg​tcg​gag​agg​cta​agc​ta
NIFK-AS1R	agg​ttg​cat​gtg​ctt​tcg​tt
USP2-AS1F	gtg​gac​tgg​aat​gtc​aca​cg
USP2-AS1R	aca​gtc​ttg​aat​cgc​tga​cg
LIPT1F	gtt​gat​ccc​gaa​cac​agg​ag
LIPT1R	ctc​att​acg​gtc​gtg​tgc​at
TOTAL	18 Primer sequences (9 genes)

### Statistical analysis

All statistical analysis and visualization were performed using R software (version 4.1.3). The R packages “ggplot2”, “ggalluvial” and “limma” were used to analyze cuproptosis related lncRNAs in TCGA breast cancer patients. Use Kaplan Meier plotter to evaluate the OS difference between the BCCuS-high and BCCuS-low risk groups. Univariate and multivariate Cox regression were constructed to assess whether the prognostic characteristics were independent of Age, Sex, Clinical Stage, and TNM stage. The above analysis is implemented using the R package “Survival”. The lasso (Least absolute shrinkage and selection operator) analysis was operated using R package “caret” and “glmnet”. The Nomogram is implemented using the “rms” R package to predict the OS. All *p*-value<.05 were considered statistically significant. The qPCR data were analyzed and mapped with GraphPad Prism8 software.

## Results

### Screening of cuproptosis related lncRNAs

**FIGURE 2:**
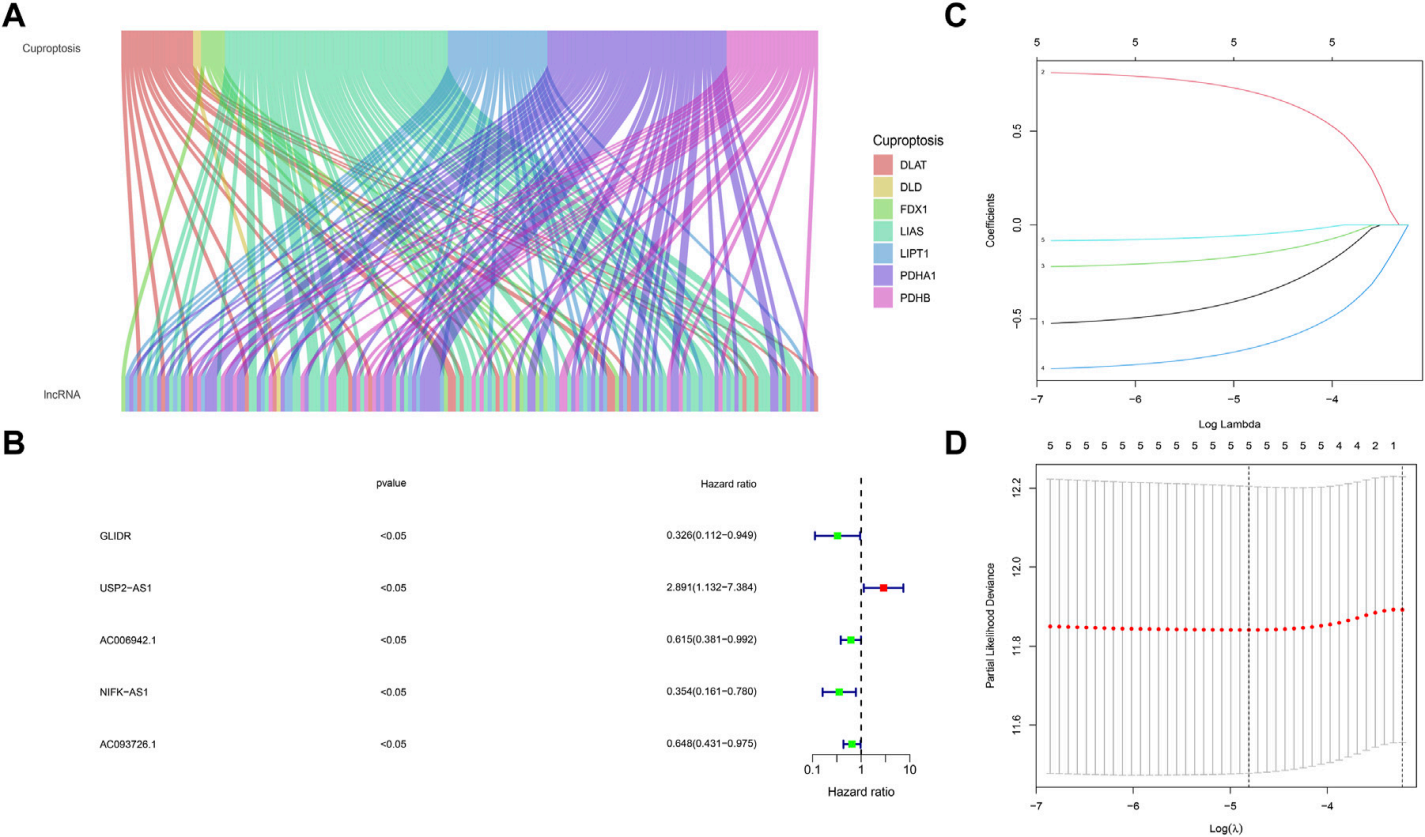
Screening of cuproptosis related lncRNAs and Prognostic value preparation **(A)** The corresponding relationship between Cuproptosis related seven genes and 153 cuproptosis related lncRNAs, the wiring indicates the corresponding relationship between mRNAs and lncRNAs, and different colors represent different cuproptosis genes **(B)** five lncRNAs (GLIDR, USP2-AS1, AC006942.1, NIFK-AS1, AC093726.1) passed Univariate COX analysis of OS (*p* < .05) **(C)** Lasso (Least absolute shrinkage and selection operator) analysis of the five lncRNAs, and two lncRNAs (USP2-AS1, NIFK-AS1) was recognized. Two lncRNAs (USP2-AS1, NIFK-AS1) were further put into model construction **(D)** Partial Likelihood Deviance of Lasso analysis.

We obtained the expression of 1,109 breast cancer samples and 103 adjacent samples from TCGA dataset. A total of 13,493 lncRNAs were screened out. The lncRNAs, which differentially expressed in breast cancer and adjacent samples obtained by Wilcox test were included in the follow-up analysis (*p* < .05). We determined the cuproptosis related lncRNAs by correlation test. Pearson correlation analysis showed that 153 lncRNAs were associated with cuproptosis related genes in breast cancer (Cor <.3, *p*-value <.05). Sankey map was used to show the corresponding relationship between Cuproptosis related seven genes and 153 Cuproptosis related lncRNAs (Figure 2A).

### Grouping fairness proof

The data of TCGA breast cancer patients were randomly divided into training set and internal verification set at a ratio of 1:1. Chi square test verified the fairness of grouping. The *p* values of chi-square test for all indicators between Test and Train were >.05, indicating the fairness of grouping.

### Establishment of cuproptosis associated lncRNA model

By using univariate cox regression analysis (*p* < .05), we identified five lncRNAs highly correlated with patients’ OS from 153 cuproptosis related lncRNAs in the training data set (Figure 2B). Then Lasso analysis screened out two genes (USP2-AS1, NIFK-AS1) with the largest relative weight for subsequent model construction (Figure 2C). Partial Likelihood deviance of Lasso analysis was also provided (Figure 2D). Next, we used the multivariate COX regression to fit cuproptosis related lncRNAs risk model (BCCuS) of breast cancer patients. The formula of prognostic risk score: BCCuS = (1.117,558 × USP2-AS1)+(−1.05486 × NIFK-AS1) (Table 2). According to the median BCCuS score, 1,109 breast cancer patients in the training data set were divided into two groups: high- BCCuS group and low- BCCuS group. In Figures 2B, C and Table 2, we can know that lncRNA USP2-AS1 is a favorable prognostic factor and lncRNA NIFK-AS1 is an unfavorable prognostic factor for breast cancer patients.

**TABLE 2:** The formula of prognostic risk score (BCCuS) fitted by multi-COX.

Model lncRNA	Multi-COX coeff
USP2-AS1	1.117,558
NIFK-AS1	−1.05486
BCCuS = (1.117,558 × USP2-AS1)+(−1.05486 × NIFK-AS1)	

### Validation of BCCuS in breast cancer

**FIGURE 3:**
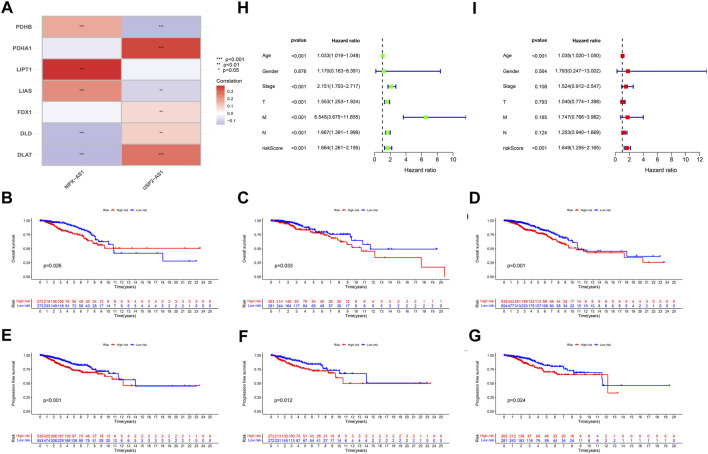
**(A)** The correlation heatmap shows the expression correlation between two lncRNA used to build the model and seven cuproptosis genes **(B)** Train set OS Kaplan Meier survival analysis between high-risk group and low-risk group **(C)** Test set OS Kaplan Meier survival analysis between high-risk group and low-risk group **(D)** TCGA-BRCA 1109 samples OS Kaplan Meier survival analysis between high-risk group and low-risk group **(E)** Train set PFS Kaplan Meier survival analysis between high-risk group and low-risk group **(F)** Test set PFS Kaplan Meier survival analysis between high-risk group and low-risk group **(G)** TCGA-BRCA 1109 samples PFS Kaplan Meier survival analysis between high-risk group and low-risk group **(H,I)** Univariate and multivariate Cox regression was constructed to assess whether the prognostic characteristics were independent of Age, Sex, Clinical Stage, and TNM stage.

**FIGURE 4:**
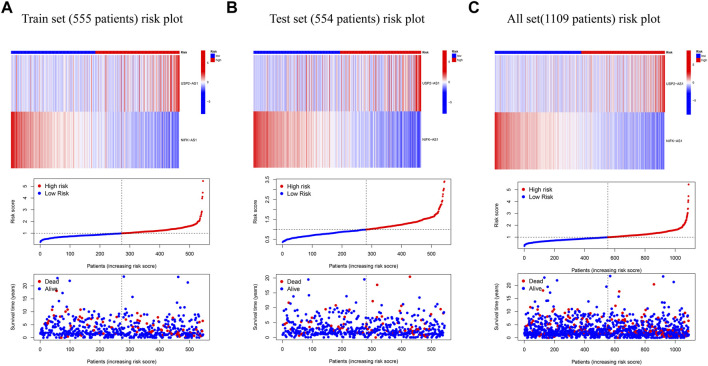
Risk plot was mapped using R package “heatmap” **(A)** Risk plot of train set (555 patients) **(B)** Risk plot of test set (554 patients) **(C)** Risk plot of TCGA-BRCA set (1,109 patients).

**FIGURE 5:**
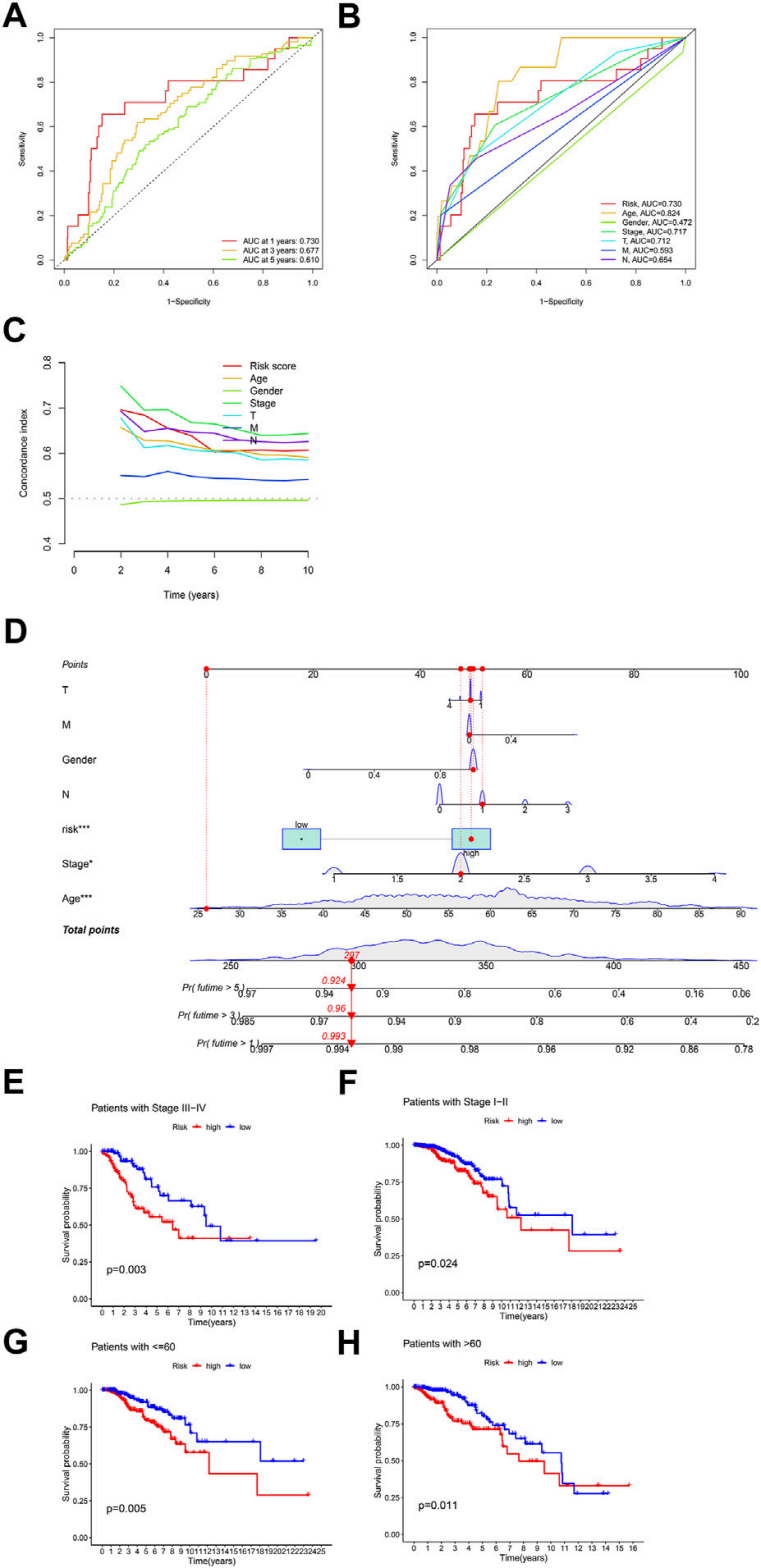
Further validation of risk model and clinical decision-making analysis **(A)** ROC curve of the risk model predicting efficiency (AUC 1-year: .730, 3-year: .677, 5-year: .610) **(B)** ROC curve contains BCCuS and other clinical factors **(C)** Concordance index of BCCuS and other clinical information **(D)** Nomogram was constructed for clinical convenient predicting of single-patient prognosis **(E,F)** Survival curve after grouping according to clinical stages (stage 1-2, stage 3-4) **(G,H)** Survival curve after grouping according to age (≤60, >60).

According to the expression data of TCGA samples, the correlation heatmap shows the expression correlation between two lncRNA used to build the model and seven cuproptosis genes (Figure 3A). USP2-AS1 was positively correlated with four genes (DLAT, PDHA1, FDX1, DLD) and negatively correlated with two genes (LIAS, PDHB). NIFK-AS1 was positively correlated with three genes (LIAS, LIPT1, PDHB) and negatively correlated with two genes (DLD, DLAT) (Figure 3A). To further evaluate the predicting ability of BCCuS in breast cancer, Kaplan Meier survival analysis was conducted in the training cohort and internal validation set from TCGA data set, based on the patient OS (overall survival) data obtained from TCGA website and the patient PFS (prognosis free survival) data obtained from XENA website (https://xena.ucsc.edu/). In train set, the results showed that the OS of breast cancer patients in the high-risk group was worse than that in the low-risk group (*p* = .026) (Figure 3B), and the PFS was also worse than that in the low-risk group (*p* < .001) (Figure 3E). In internal validation set, the OS of breast cancer patients in the high-risk group was worse than that in the low-risk group (*p* = .033) (Figure 3C), as well as PFS (*p* = .012) (Figure 3F). In all TCGA-BRCA 1109 samples, the OS of breast cancer patients in the high-risk group was worse than that in the low-risk group (*p* = .001) (Figure 3D), as well as PFS (*p* = .024) (Figure 3G). Univariate and multivariate Cox regression were constructed to assess whether the prognostic characteristics were independent of age, sex, clinical Stage, and TNM stage (Figures 3H,I). Results show that BCCuS might be an independent prognostic factor and could partially eliminate the interference of clinical factors. Risk plot was mapped using R package “heatmap” and in both sets, USP2-AS1 was appeared to express higher in BCCuS-high group, NIFK-AS1 express higher in BCCuS-low group, which shows the reliability of the model gene (Figures 4A–C). Further validation of risk model was conducted using c to check the predicting efficiency, with AUC (Area Under Curve): 1-year: .730, 3-year: .677, 5-year: .610 (Figure 5A). ROC curve contains BCCuS, and other clinical factors shows BCCuS was a better predicting signature than clinical stage (BCCuS AUC = .730, stage AUC = .717) (Figure 5B). Concordance index of BCCuS and other clinical information indicated that BCCuS could be a reliable clinical reference index (Figure 5C). The nomogram was constructed based on the score for clinical convenient predicting of single-patient prognosis (Figure 5D). Further, KM-plot was mapped after grouping of overall samples according to clinical stages (stage 1-2: *p* = .024; stage 3-4: *p* = .003), and according to age (≤60: *p* = .005; >60: *p* = .011) (Figures 5E–H).

### Model related biological mechanism

**FIGURE 6:**
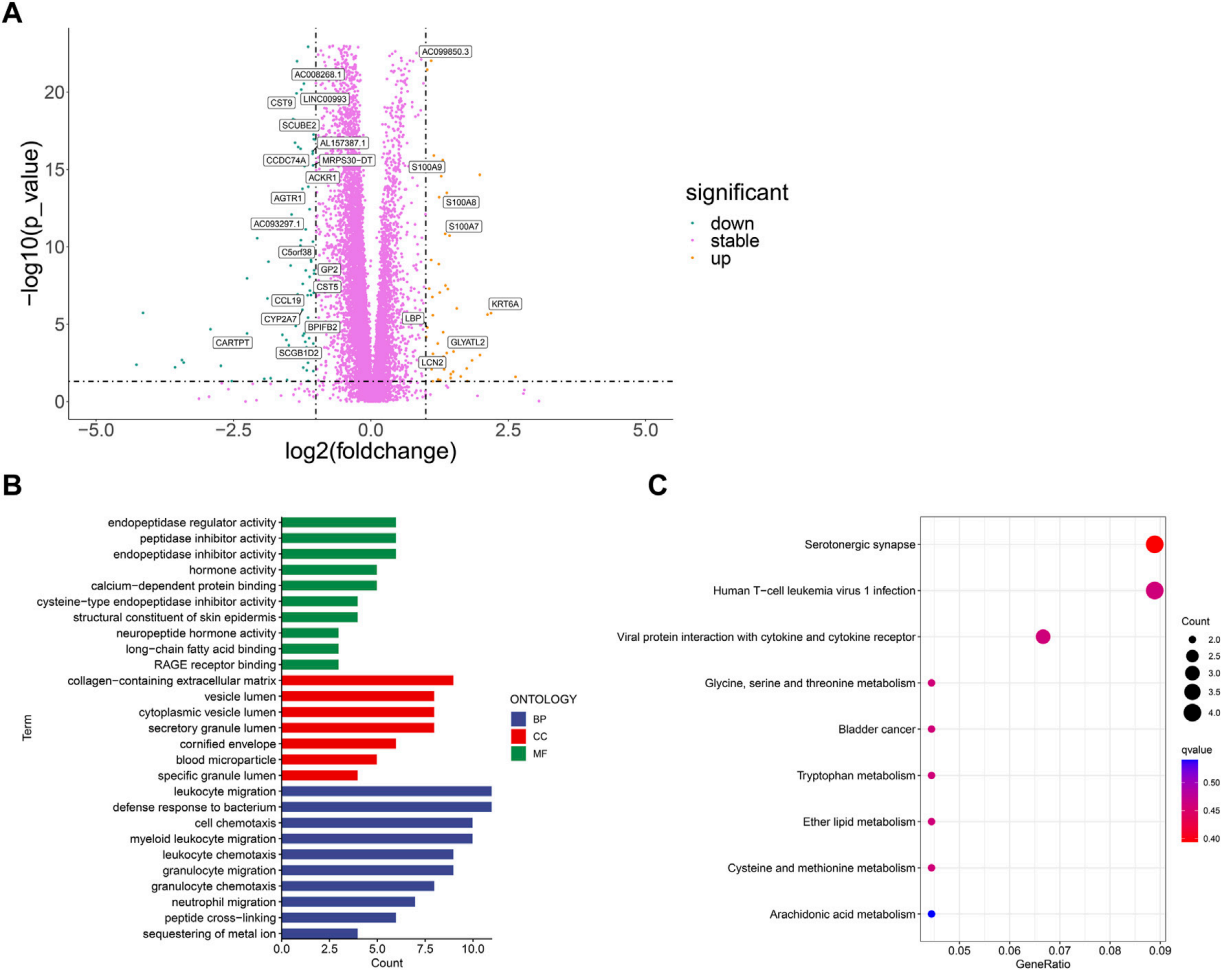
Correlation between risk score and biological mechanism **(A)** Volcano plot displays significantly expressed genes between high-risk and low-risk groups (*p* < .001, |log_2_Foldchange|>1) **(B)** GO analysis shows Enriched terms **(C)** KEGG analysis shows Enriched pathways.

First, we used limma package to analyze the differentially expressed genes between BCCuS-high and BCCuS-low group (*p* < .001, |log2Foldchange|>1) (Figure 6A). 26 differential genes are screened out and displayed using volcano plot. Among these 26 genes, 18 genes down-regulated, and eight genes up-regulated, including calcium binding proteins S100A8 and S100A9. S100A8/A9 expresses in both the tumor microenvironment and the external environment and can be classified as a signal during the tumorigenesis (Moon et al., 2008; Woo et al., 2021). GO enrichment analysis reveals that differentially expressed genes enriched in Metabolic metabolism related molecular function and immune response (Ashburner et al., 2000), such as sequestering of metal ion and leukocyte migration (Figure 6B). KEGG results shows that BCCuS-high and BCCuS-low group enriched differentially in pathways related to immune response and energy metabolism, for example, serotonergic synapse and viral protein interaction with cytokine receptor (Figure 6C) (Kanehisa et al., 2017).

### Relationship between BCCuS and immune function

**FIGURE 7:**
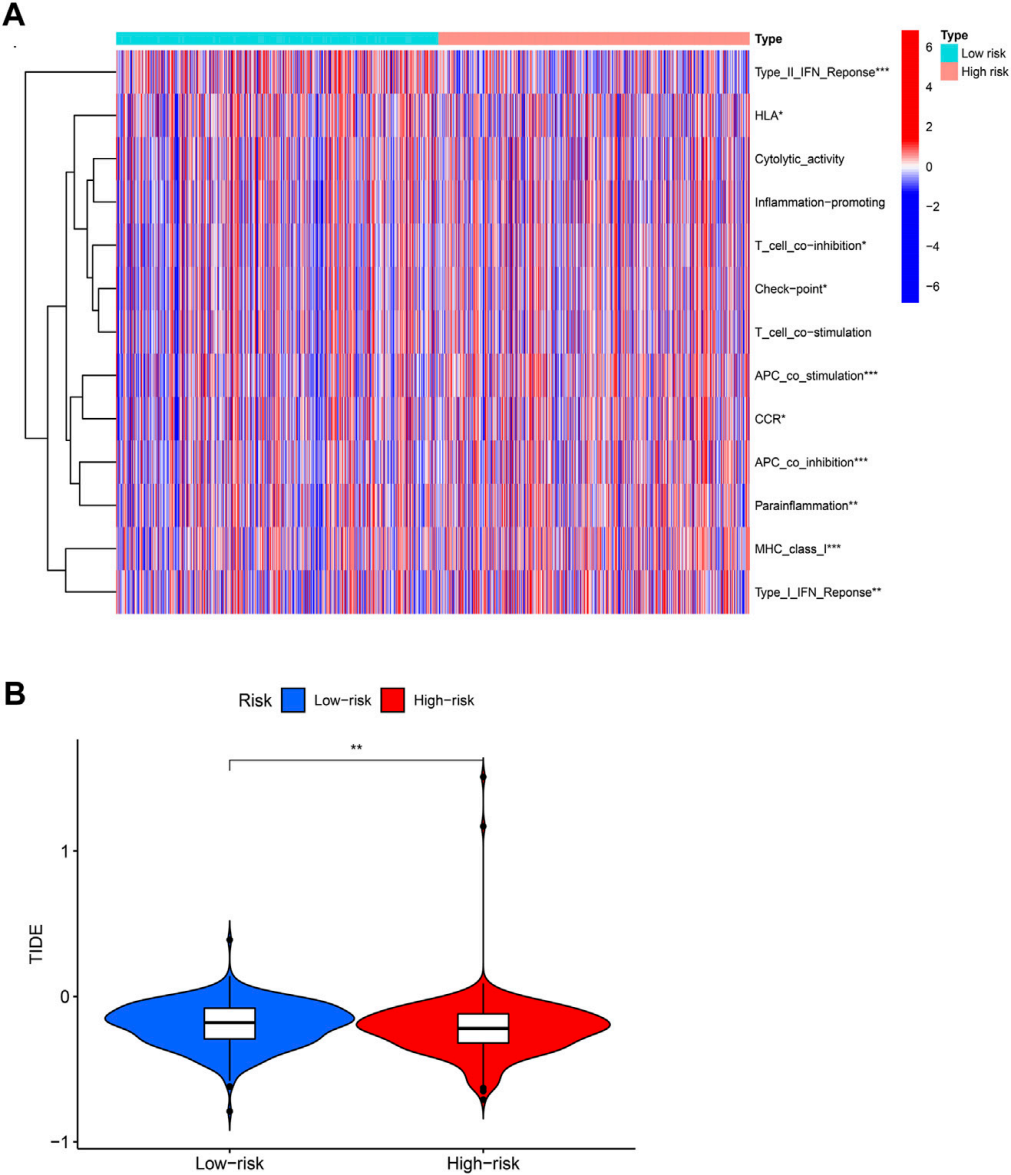
Relationship between score and immunity **(A)** Using R package “GSVA” to calculate the significant difference enrichment of immunological function between high and low risk groups (*p*-value < .001: “***”, *p*-value < .01: “**”, *p*-value < .05: “*”) **(B)** BCCuS-high and BCCuS-low group show significant difference in tumor immune dysfunction and exclusion (TIDE) score (https://tide.dfci.harvard.edu/).

We use R package “GSVA” to calculate the significant difference enrichment of immunological function between high and low risk groups. Only type II IFN response was significantly inhibited in BCCuS-high group. HLA, T cell co-inhibition, check point, APC co-stimulation, CCR, APC co-inhibition, Para inflammation, MHC class I and Type I IFN response all significantly promoted in BCCuS-high group (*p* < .05) (Figure 7A). Tumor immune dysfunction score (TIDE) is consistent with tumor immune escape characteristics and can predict the effect of immunosuppression therapy (Jiang et al., 2018). BCCuS-high group shows significantly lower TIDE score, which may reveal the immune escape of tumor cells and may lead to poor prognosis (Figure 7B).

### Relationship between model score and mutation

**FIGURE 8:**
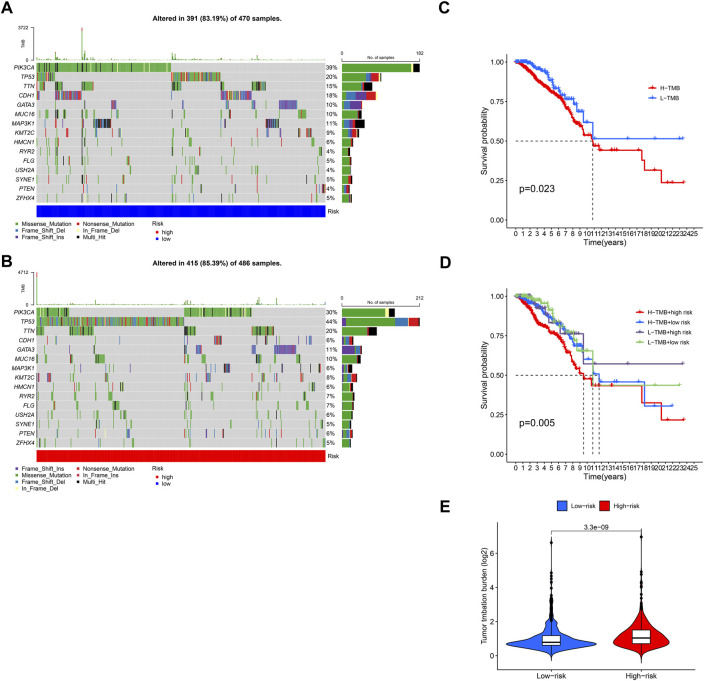
Relationship between model score and mutation **(A,B)** 1,109 Breast cancer samples were divided into high and low groups according to the median risk scores, and the frequency of gene mutations and TMB in the two groups was calculated by using “maftools” R package **(C)** TMB (tumor mutation burden) data and base mutation data of breast cancer samples were downloaded from TCGA database. KM plot was mapped between TMB-high and TMB-low groups **(D)** According to BCCuS and TMB, TCGA samples were divided into four groups, and survival curve was fitted **(E)** Low-risk and high-risk group show significant differences in TMB.

1,109 Breast cancer samples were divided into high and low groups according to the median risk scores. Frequency of mutations and TMB in both groups were calculated by using “maftools” R package. In BCCuS-low group, the mutation frequency of PIK3CA, CDH1, MAP3K1 up-regulated, these genes have been reported as tumor suppressor in breast cancer (Figure 8A) (Jiang et al., 2014; Couch et al., 2017; Ge et al., 2017; Zacksenhaus et al., 2017; Xue et al., 2018; Wijshake et al., 2021). While TP53 and TTN, were upregulated in BCCuS-high group, functioning as a promoter in breast cancer (Figure 8B) (Zhang et al., 2018a; Mao et al., 2018; Badve and Gökmen-Polar, 2019; Zheng et al., 2021). TMB (tumor mutation burden) data and base mutation data of breast cancer samples were downloaded from TCGA database. KM plot was mapped between TMB-high and TMB-low groups and TMB-high shows lower survival probability (*p* = .023) (Figure 8C). Then according to BCCuS and TMB, TCGA samples were divided into four groups, and survival curve was fitted. BCCuS-high and TMB-high group shows the highest risk (*p* = .005) (Figure 8D). Violin plot reveals that high-risk group has close ties with higher TMB (Figure 8E).

### Machine learning screening of BCCuS-sensitive anticancer drugs

**FIGURE 9:**
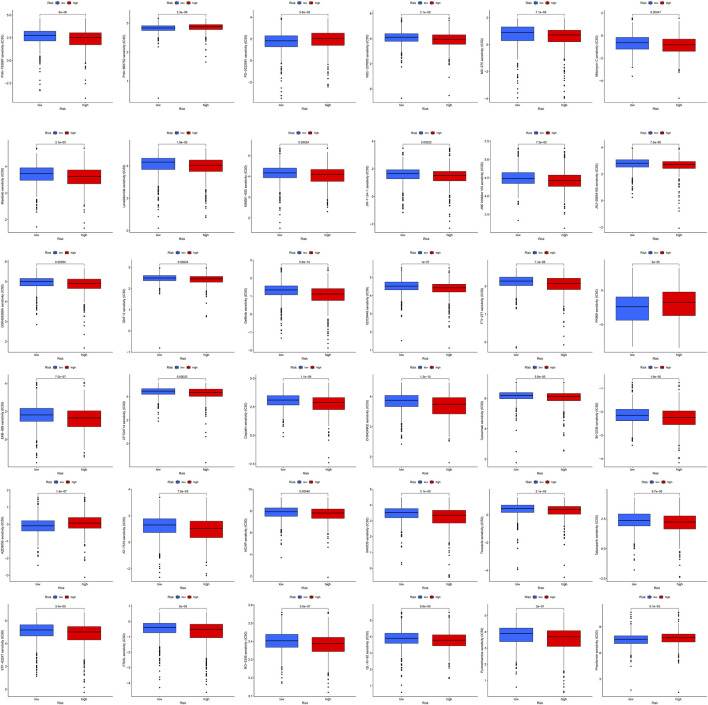
By using pRRophetic package and machine learning, 36 anticancer drugs whose sensitivity (IC50) was significantly related to the model were screened out. The threshold of *p*-value was set to .001, and the anticancer drug sensitivity database used was CPG 2016.

By using pRRophetic package and machine learning (Geeleher et al., 2014), 36 anticancer drugs whose sensitivity (IC50) was significantly related to the model were screened out. The threshold of *p*-value was set to .001. It is worth noting that several kinds of tumor chemotherapy drugs and targeted drugs have higher sensitivity in high-risk group, for example, Cisplatin, Masitinib, Gefitinib, and Tivozanib (Figure 9). Among 36 anticancer drugs, 31 had higher sensitivity in high BCCuS group, while five had lower sensitivity in high BCCuS group.

**FIGURE 10:**
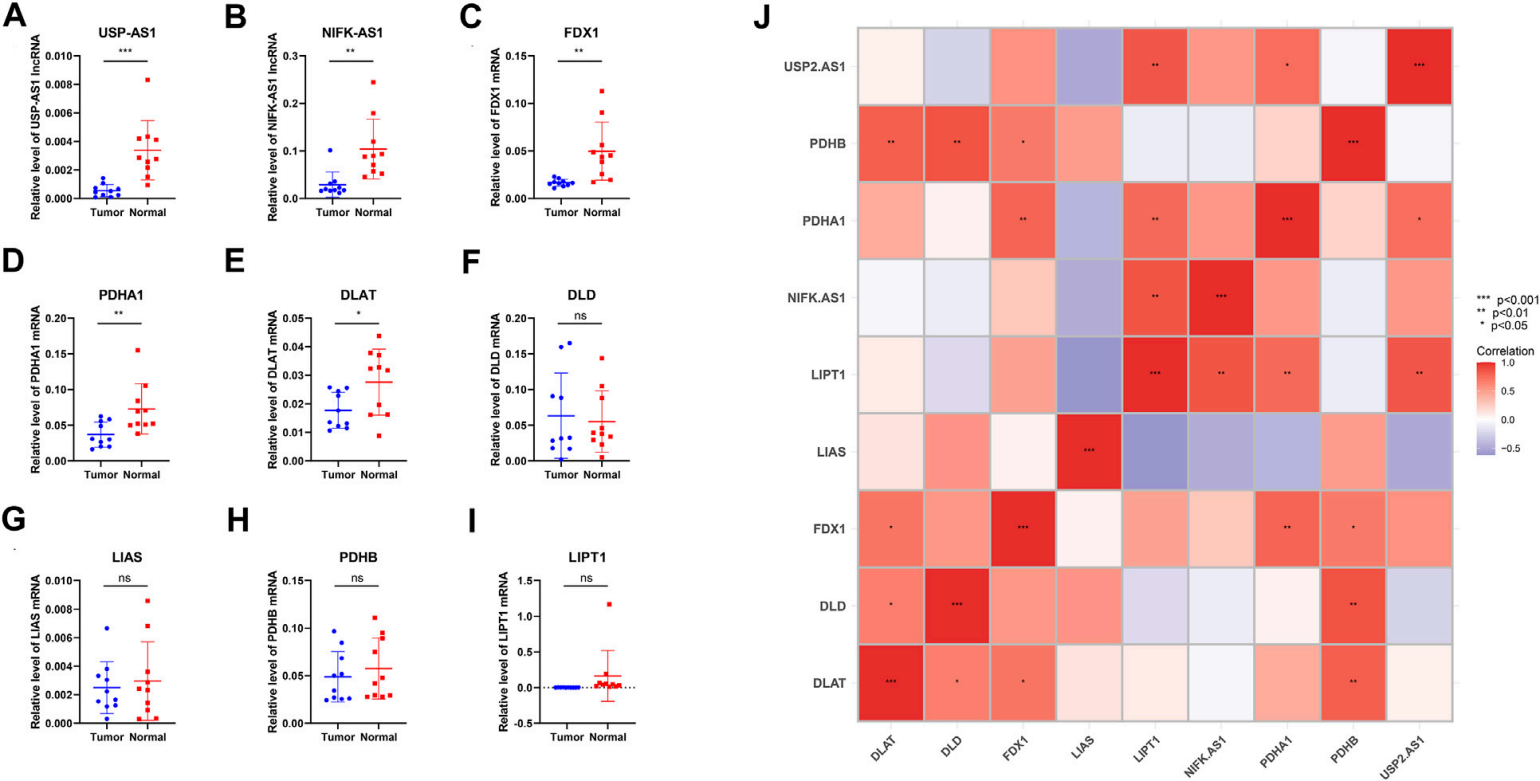
qPCR of primary breast cancer tissues and normal tissues adjacent to the cancer from 10 patients with breast cancer diagnosed in the breast center of Jiangsu Province, China **(A,B)** qPCR relative expression data of two model lncRNAs **(C–I)** qPCR relative expression data of seven cuproptosis-related genes **(J)** Correlation heat map of nine Cuproptosis-related genes and two model lncRNAs’ experimental expression (*p*-value < .001: “***”, *p*-value < .01: “**”, *p*-value < .05: “*”).

### Experimental validation by qPCR

qPCR of primary breast cancer tissues and normal tissues adjacent to the cancer from 10 patients with breast cancer were operated. Results illustrated that two model lncRNAs both differentially expressed in cancer and Para cancerous samples, USP2-AS1 and NIFK-AS1 both had lower expression in cancer samples. FDX1, PDHA1 and DLAT also significantly down regulated in breast cancer. Correlation heatmap of seven cuproptosis-related genes and two model lncRNAs was constructed using experimental expression. The result shows the experimental expression of USP2-AS1 was positively correlated with PDHA1, NIFK-AS1 was positively correlated with LIPT1, this is consistent with the previous bioinformatic prediction.

## Discussion

Cuproptosis is a newly defined regulated form of cell death that is mediated by the accumulation of copper ions in cells. Dozens of enzymes in the human body contain copper ions or use copper ions. Copper ions can provide or receive electrons, thus catalyzing key biochemical reactions. Tumor is particularly dependent on copper ions (Zhang et al., 2018b; Xu et al., 2022b; Zheng et al., 2022). Enzyme containing copper ions, such as lysyl oxidase like two proteins (LOXL2), can produce collagen scaffold structures and help cancer cells migrate (Shanbhag et al., 2019). In the clinical trial of treating breast cancer patients with copper ion chelators, the level of LOXL2 was reduced. It has been confirmed that there is a relationship between copper metabolism and metastasis of breast cancer. For example, the copper binding protein Atox1 drives cell movement by stimulating the transport chain composed of another copper transporter, ATP7A, and lysyl oxidase (LOX). However, the combined effect of lncRNA and copper ion in breast cancer has not been reported.

Therefore, our work identified 153 lncRNAs related to cuproptosis in breast cancer and screened two lncRNAs (USP2-AS1, NIFK-AS1) to construct risk model, abbreviated as BCCuS. This model has been validated by internal validation set and could be an independent prognostic factor for breast cancer patients. We further constructed a nomogram to predict the OS of clinical patients. Subsequently, the patients were divided into two groups according to the median risk score as the cutoff value. Through KEGG and GO analysis, we found that the model was significantly related to immune and metabolic pathways. At the same time, the two groups showed significant differences in the mutation frequency of high-frequency mutation genes. The mutation frequency of oncogenes in breast cancer such as TP53 and TTN was higher in the high-risk group, while the mutation frequency of tumor suppressor factors in breast cancer such as PIK3CA, CDH1, MAP3K1 was low in the high-risk group.

By using pRRophetic package and machine learning, 36 anticancer drugs whose sensitivity (IC50) was significantly related to the model were screened out. 31 anticancer drugs had lower IC50 and higher sensitivity to high BCCuS group, such as Cisplatin, Masitinib, Gefitinib. Cisplatin has been widely used in chemotherapy of breast cancer (Ezzat et al., 1997; Qin et al., 2018; Wang et al., 2021). Gefitinib was proved to be effective in the treatment of hormone resistant and hormone receptor negative advanced breast cancer in the phase II clinical trial (Polychronis et al., 2005; Green et al., 2009). Masitinib has not been reported in treating breast cancer, further cell line drug sensitivity experiments can be conducted. Five anticancer drugs had higher and lower sensitivity to high BCCuS group, such as Phenformin, which can improve insulin sensitivity of breast cancer tissue, consuming nucleotide triphosphate and may hinder nucleotide synthesis, thus playing a role in inhibiting cancer (Janzer et al., 2014).

Our current research only uses TCGA data, and data validation from other sources needs to be supplemented in subsequent work. In qPCR expression of 10 pairs of breast cancer and adjacent samples, FDX1, key upstream gene of cuproptosis pathway, downregulated in cancer samples. Cuproptosis could act as a protective factor in normal body cells, for initiating programmed cell death in cells with abnormal accumulation of copper ions. In breast cancer cell, lack of FDX1 may lead to the failure in startup of this “suicide” mechanism, leading to the escape of breast cancer cells from cuproptosis. Two model lncRNAs, USP2-AS1 and NIFK-AS1 both downregulated in breast cancer samples, while USP2-AS1 is a risk factor in uniCox regression. USP2-AS1 is reported to be a direct transcriptional target of the oncoprotein c-Myc, and the expression levels of c-Myc and USP2-AS1 are positively correlated in different types of cancer, including colon adenocarcinoma (COAD), rectal adenocarcinoma (READ), breast cancer invasive carcinoma (BRCA), prostate adenocarcinoma (PRAD) and gastric adenocarcinoma (STAD), therefore we can determine USP2-AS1 as the cancer promoting factor of breast cancer (Li et al., 2021). Different from other cancers, c-Myc is up-regulated in only one-third of breast cancer, so the lower expression of USP2-AS1 in breast cancer can be explained, insufficient sample size may also increase bias (Beroukhim et al., 2010). In univariate Cox analysis, one-third of patients with up-regulated expression of USP2-AS1 may contribute to a particularly short survival time, which ultimately leads to a statistically determined risk factor. Subsequent research will focus on distinguishing the subtypes with up regulation and down regulation of c-Myc in breast cancer, and studying the prognostic role of USP2-AS1 in subtype samples.

Overall, our study constructed a novel cuproptosis-related lncRNA signature (BCCuS) in breast cancer. And we experimentally verified the cuproptosis gene set and model lncRNAs were.

## Conclusion

Taken together, our study defined a novel cuproptosis-related lncRNA signature (BCCuS) in breast cancer. The cuproptosis-related lncRNA signature may provide new insights into predicting the prognosis of patients with breast cancer. The related pathways and immunological functions of BCCuS may help to develop new therapeutic targets for breast cancer. The screening of anti-cancer drugs sensitive in both high and low BCCuS groups may be able to improve the benefit rate of patients.

## Statistical analysis

Statistical analyses were performed using the R v.4.1.3 (https://www.r-project.org/). The linear mixed-effects model was used to analyze the differences in gene expression between tumor and normal tissues. Univariate and multivariate Cox regression analyses or the Log-ranch test were used to investigate the relationship between gene expression and the patients’ overall survival and to construct the risk model. The association between risk model and the immune function, as well as drug sensitivity, was given *via* the calculation of Spearman’s or Pearson’s correlation coefficients. In addition, linear regression was used to investigate the relationship between gene expression and the patients’ clinical characteristics, immune components, TIDE score, and TMB. Statistical significance is defined as *p* < .05. The relative gene expression in PCR was obtained by logarithmic conversion of CT value.

## Data Availability

The original contributions presented in the study are included in the article/supplementary material, further inquiries can be directed to the corresponding authors.

## Abbreviations

BCCuS, cuproptosis-related lncRNA signature in breast cancer; lncRNA, Long-noncoding RNA; TCGA, The Cancer Genome Atlas; TIDE, tumor immune dysfunction and exclusion; TMB, tumor mutation burden; CLEOPATRA, Clinical Evaluation of Pertuzumab and Trastuzumab; TCA, tricarboxylic acid cycle; qRT-PCR, Quantitative real-time PCR; OS, overall survival; AUC, Area Under Curve; BRCA, breast cancer; LOX, lysyl oxidase; LOXL2, lysyl oxidase like 2 proteins.
